# Ticks harbor and excrete chronic wasting disease prions

**DOI:** 10.1038/s41598-023-34308-3

**Published:** 2023-05-15

**Authors:** H. N. Inzalaco, F. Bravo-Risi, R. Morales, D. P. Walsh, D. J. Storm, J. A. Pedersen, W. C. Turner, S. S. Lichtenberg

**Affiliations:** 1 Wisconsin Cooperative Wildlife Research Unit, Department of Forest and Wildlife Ecology, University of Wisconsin, Madison, Madison, WI 53706 USA; 2 Department of Neurology, The University of Texas Health Science Center at Houston, Houston, TX USA; 3 Centro Integrativo de Biologia y Quimica Aplicada (CIBQA), Universidad Bernardo O’Higgins, Santiago, Chile; 4 U.S. Geological Survey, Montana Cooperative Wildlife Research Unit, University of Montana, Missoula, MT USA; 5 Wisconsin Department of Natural Resources, Eau Claire, WI USA; 6 Environmental Health and Engineering, Johns Hopkins University, Baltimore, MD 21218 USA; 7 Wisconsin Cooperative Wildlife Research Unit, Department of Forest and Wildlife Ecology, U.S. Geological Survey, University of Wisconsin – Madison, Madison, WI 53706 USA; 8 Department of Soil Science, University of Wisconsin, Madison, Madison, WI USA

**Keywords:** Ecology, Infectious diseases

## Abstract

Chronic wasting disease (CWD) is a fatal neurodegenerative disease caused by infectious prions (PrP^CWD^) affecting cervids. Circulating PrP^CWD^ in blood may pose a risk for indirect transmission by way of hematophagous ectoparasites acting as mechanical vectors. Cervids can carry high tick infestations and exhibit allogrooming, a common tick defense strategy between conspecifics. Ingestion of ticks during allogrooming may expose naïve animals to CWD, if ticks harbor PrP^CWD^. This study investigates whether ticks can harbor transmission-relevant quantities of PrP^CWD^ by combining experimental tick feeding trials and evaluation of ticks from free-ranging white-tailed deer (*Odocoileus virginianus*). Using the real-time quaking-induced conversion (RT-QuIC) assay, we show that black-legged ticks (*Ixodes scapularis*) fed PrP^CWD^-spiked blood using artificial membranes ingest and excrete PrP^CWD^. Combining results of RT-QuIC and protein misfolding cyclic amplification, we detected seeding activity from 6 of 15 (40%) pooled tick samples collected from wild CWD-infected white-tailed deer. Seeding activities in ticks were analogous to 10–1000 ng of CWD-positive retropharyngeal lymph node collected from deer upon which they were feeding. Estimates revealed a median infectious dose range of 0.3–42.4 per tick, suggesting that ticks can take up transmission-relevant amounts of PrP^CWD^ and may pose a CWD risk to cervids.

## Introduction

Chronic wasting disease (CWD) is an infectious, slowly progressing, and invariably fatal neurodegenerative disease afflicting wild and domestic cervids. Both direct and indirect routes of exposure and transmission have contributed to endemic increases and broad geographic spread of CWD^1^. However, there are significant gaps in our understanding of how CWD is transmitted among susceptible hosts. Proposed putative routes of exposure and transmission among susceptible cervid species include sexual contact^2^, consumption of contaminated soil, water, and plants^3–5^, mucosal contact with contaminated fomites^6^, or antler cannibalism behavior^7^. With many unknowns about transmission pathways and their relative risks it is prudent to consider how host behavior and life-history traits facilitate CWD exposure events. Here we examine the potential for ticks to contribute to indirect transmission of CWD.

In the host, CWD presents with a broad distribution of disease-associated prions (PrP^CWD^) in peripheral tissues and biological fluids prior to neuroinvasion. Blood of prion-infected animals harbors infectivity at the pre-symptomatic disease stage with relatively higher circulating amounts of PrP^CWD^ than those found in urine^8^ or feces^9^. Animal challenge studies demonstrate that CWD-positive whole blood has a disease attack rate of up to 100% in cervids following intravenous exposure with 250 mL and 22% in cervidized mice following oral exposure with 150 µL^10^.

Arthropods that interact with cervids, such as biting flies or blood-obligate ectoparasites, could play a role in prion transmission. Early investigations revealed that homogenates of several species of mites gathered from scrapie infected sheep farms harbored infectivity following intracerebral (i.c.) and intraperitoneal (i.p.) exposure of mice^11^. Recent studies examining the role of ticks in transmission of transmissible spongiform encephalopathies (TSEs) suggest that nymphal ticks would be poor mechanical vectors for certain disease-causing prions, but that adults of at least one species of Ixodid tick may have the potential to take up PrP^CWD^^12,13^. Ticks possess several biological and behavioral traits that may implicate their involvement in indirect transmission. Ticks take a blood meal that can range in volume from 0.3 mL to as high as 8.9 mL per female^14^. Ixodid ticks remain attached to one bite location for as long as 14 days^14^, during which time there is a rapid engorgement phase in the last 24–36 h where consumed blood is concentrated due to reduced digestion and excretion of water and electrolytes^15^. This concentration of blood meal results in a fed body weight increase in excess of 100 times their unfed weight^16^. This tick feeding behavior may be pertinent to disease transmission since it may concentrate infectious prions.

Arthropods and other invertebrates do not express cellular prion (PrP^C^), a prerequisite for establishing a prion infection^17^, indicating that a more likely role for prion disease transmission by ticks would be as a mechanical vector rather than as a biological vector. Cervids may encounter ticks harboring prion infected blood during bouts of allogrooming, an ectoparasite-defense strategy used by social mammals^18^ that involves grooming between members of the same species. This form of grooming is one of the most common nonaggressive interactions among females, females and young, as well as among males during the non-mating season of several cervid species^18,19^. Higher ectoparasite infestations increase allogrooming behavior in cervids such as white-tailed deer (*Odocoileus virginianus*, WTD)^19^ and elk (*Cervus canadensis*)^18^, during which ectoparasites may be consumed intentionally or unintentionally, as a result of grooming mechanics such as licking, or nibbling and chewing^19^. These host and parasite traits may make allogrooming a possible transmission pathway for CWD, if hosts consume partially or fully engorged ticks during bouts of allogrooming.

Using an ultrasensitive in vitro protein amplification assay, real-time quaking-induced conversion (RT-QuIC), we investigate this hypothesized pathway to (i) determine experimentally if ticks can harbor prions taken up from infected blood meals and (ii) survey ticks on CWD-positive WTD to determine if PrP^CWD^ can be detected in ticks collected from free-ranging deer in a CWD endemic region. Given that prion seeding activity was detected in these ticks, we further (iii) estimated amounts of PrP^CWD^ found in ticks relative to amounts of PrP^CWD^ found in CWD-positive deer lymphatic tissues, and (iv) estimated a per-tick infectious dose (ID_50_) based on the pooled tick amyloid formation rate (AFR) (i.e., 1/time to threshold) equivalence to AFRs of retropharyngeal lymph node (RPLN) combined with an established minimum mass of CWD-positive brain sufficient to orally transmit CWD.

## Results

**Figure 1:**
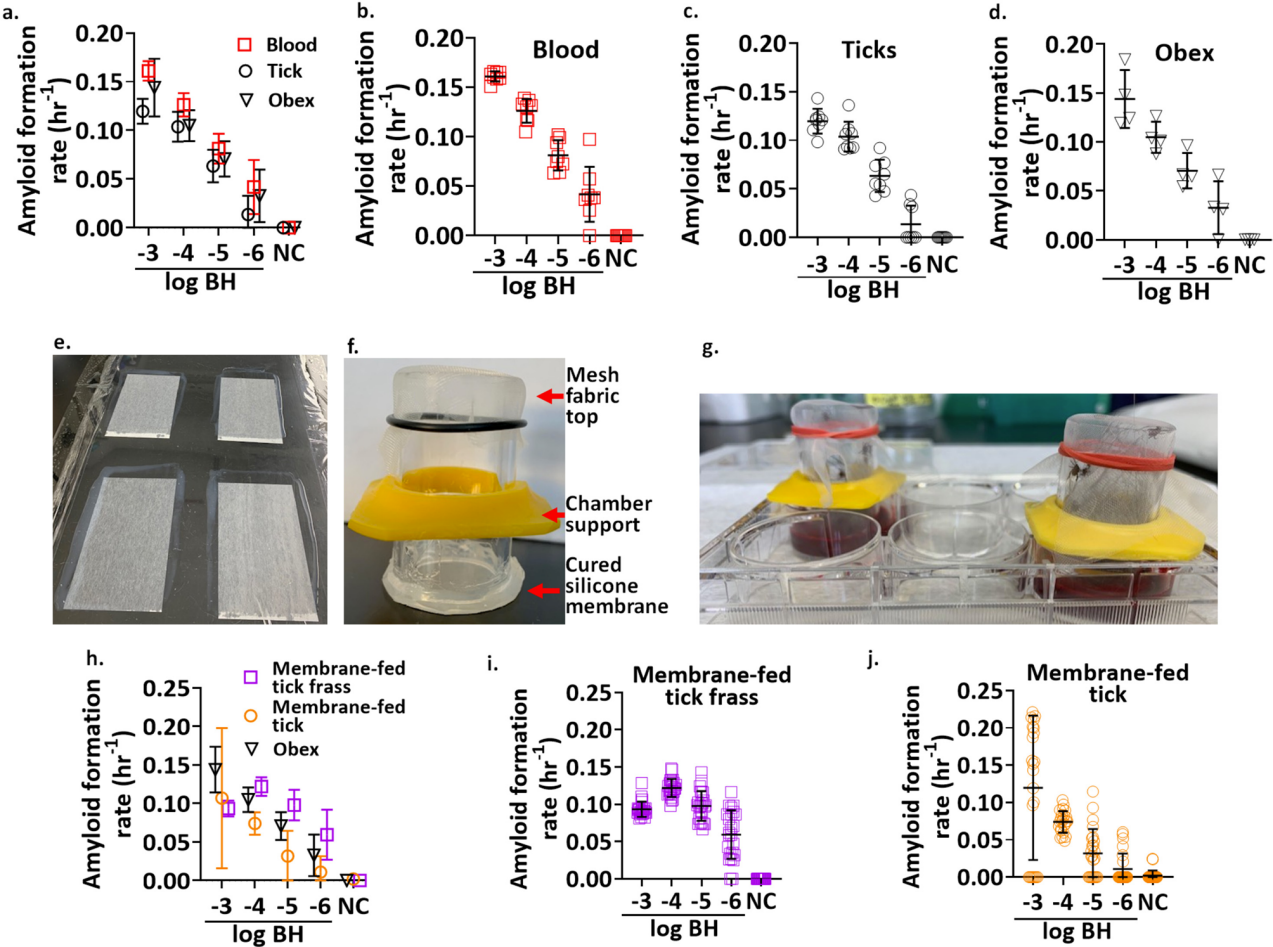
Recovery of chronic wasting disease (CWD) prions (PrP^CWD^) from spiking and membrane feeding experiments for analysis using the real-time quaking-induced conversion (RT-QuIC) assay. Comparison of amyloid formation rates (AFR) by RT-QuIC of (**a**–**c**) defibrinated bovine whole blood spiked with a 10^–3^ dilution of the same 10% brain homogenate as used for the brain dilution series, and artificial membrane-fed tick homogenates spiked in the same manner as the blood (“b” and “c” depict AFRs of all 8 technical replicates for spiked whole blood or tick homogenates averaged in “a”) or (**d**) tenfold dilutions of 10% brain homogenate (BH) (from the obex region) from a CWD-positive white-tailed deer. Membrane feeding units were constructed using (**e**) cured silicon membranes adhered to the base of (**f**) assembled feeding chambers. (**g**) Depiction of the assembled feeding unit with feeding chambers being held upright by the chamber supports. (**h**-**j**) Comparison of AFRs by RT-QuIC of homogenates from membrane-fed PrP^CWD^ exposed or negative control ticks and tick frass (“i “and “j” depict AFRs for all 24 technical replicates (from 3 biological replicates run on 3 separate plates) for frass or membrane-fed tick homogenates averaged in “h”). Negative controls (NC) in each AFR plot are representative for the same sample type.

Our experimental spiking studies aimed to test recovery of PrP^CWD^ from different sample types, using a brain sample (from the obex region) from a late-stage CWD-positive WTD as the source material for spiking in all experiments. We were able to detect PrP^CWD^ using RT-QuIC from samples spiked with PrP^CWD^, including blood, tick homogenates and homogenates of ticks fed blood spiked with PrP^CWD^. Whole blood or tick homogenates spiked with tenfold dilutions of CWD-positive brain homogenates showed sensitivity of at least 10^–6^, which corresponds with the sensitivity detection limit for the brain sample used for the spiking experiments (Fig. 1a,b,c,d). Blood and engorged tick homogenates spiked with 10^–3^ through 10^–5^ dilutions of CWD-positive brain showed PrP^CWD^ seeding activity (assay fluorescence from sample with PrP^CWD^ present) in 8/8 technical replicates (Fig. 1a,b,c). The blood and tick homogenates spiked with the 10^–6^ dilution of the CWD-positive brain showed PrP^CWD^ seeding activity in 7/8 and 4/8 replicates, respectively (Fig. 1a,b,c). Neither blood nor tick homogenate sample types produced any false seeding activity, constituting a specificity rate of 100% for each sample type using the RT-QuIC assay in these spiking experiments (Fig. 1a). AFR values for brain-spiked samples differed among sample types (*F* (2, 68) = 18.626, *p* < 0.0001), with mean AFR values higher for blood (mean ± standard error: 0.1 ± 0.003), compared with brain (0.09 ± 0.004), or tick homogenates (0.08 ± 0.003; Tukey honestly significant difference (HSD) post-hoc test blood versus brain: 0.01 ± 0.006, *p* = 0.03; blood versus tick: 0.2 ± 0.005 *p* < 0.001). AFR values for all sample types decreased across the dilution series (*F* (3, 68) = 131.352, *p* < 0.0001), however, there was no statistically significant interaction between AFR values by sample type across the dilution series (*F* (6, 68) = 0.871, *p* > 0.05).

To evaluate whether ticks can take up and excrete prions, we used a previously established artificial tick membrane-feeding system^20^ to feed *I. scapularis* a blood meal inoculated with a 10^–3^ dilution of CWD-positive brain (10^6^ ng) (Fig. 1e,f,g). Although, the relative amounts of PrP^CWD^ found in blood of CWD-infected deer is likely ~ 1000-fold less than the dilution used to inoculate blood meals^21^, this 10^–3^ mg/mL dilution was chosen to demonstrate the potential for *I. scapularis* to assimilate prions from a blood meal rather than demonstrate natural uptake. Feeding assay attachment rates reached 100% by 72 h following placement of adult female and male ticks within blood-exposed feeding chambers for all treatment groups and individual engorgement occurred between day 9 and 14 across both treatment groups. Serial dilutions of tick homogenates from the 10^–3^ PrP^CWD^ membrane-fed exposure group demonstrated PrP^CWD^ seeding activity in 15/24, 24/24, 15/24, and 2/24 technical replicates for inoculum-based dilutions of 10^–3^ through 10^–6^, respectively (Fig. 1h,j). Serial dilutions of tick frass collected from the 10^–3^ PrP^CWD^ treatment group demonstrated seeding activity in 24/24 replicates for inoculum-based dilutions of 10^–3^ through 10^–5^, and 22/24 replicates for the 10^–6^ inoculum-based dilution (Fig. 1h,i). AFR values were significantly different based on sample type (*F* (2, 196) = 24.753, *p* < 0.0001). Mean AFRs were significantly higher for frass (mean ± standard error: 0.093 ± 0.0038), and brain (0.088 ± 0.0094), compared to tick homogenates (0.06 ± 0.004; Tukey HSD post-hoc test of frass versus tick: 0.04, ± 0.005, *p* < 0.0001; brain versus tick: 0.03, ± 0.010, *p* = 0.005). AFR values differed along the dilution series (*F* (3, 196) = 24.848, *p* < 0.0001), with a significant interaction between sample type and dilution (*F* (6, 196) = 5.759, *p* < 0.0001; significant Tukey HSD post-hoc test: frass and brain for the 10^–3^ dilution: − 0.05, ± 0.02, *p* = 0.036; frass and tick at dilutions 10^–4^ to 10^–6^: + 0.05, ± 0.01, *p* < 0.0001; 0.07, ± 0.01, *p* < 0.0001; 0.05, ± 0.01, *p* < 0.0001, respectively).

After these proof-of-concept experiments, we examined ~ 2000 Wisconsin hunter-harvested deer heads for ticks. Of the 2000 heads examined, 174 were tick-infested. From the sample set of 174 heads infested with ticks that were evaluated for CWD in RPLN through enzyme-linked immunosorbent assay (ELISA), 15 tested positive (data not shown). CWD status in these heads were cross-confirmed by RT-QuIC, providing similar results. Then, we determined if prions could be detected in ear tissue and in engorged ticks from these 15 CWD-positive WTD (See Supplementary Table S1 for county harvested in). As negative control, 15 additional pooled tick samples collected from CWD-negative WTD were included in this analysis but were analyzed without blinding in completely separate experiments (Supplemental Figures S1 and S2). The number of attached and partially or fully engorged ticks collected from each WTD head examined, regardless of CWD status, ranged from 1 to 30 (6.1 ± 5.4). The number of attached ticks ranged from 2 to 8 (3.7 ± 1.8) for the 15 CWD-positive deer heads and 3 to 16 (5.3 ± 4.4) for the 15 CWD-negative deer heads.

**Figure 2:**
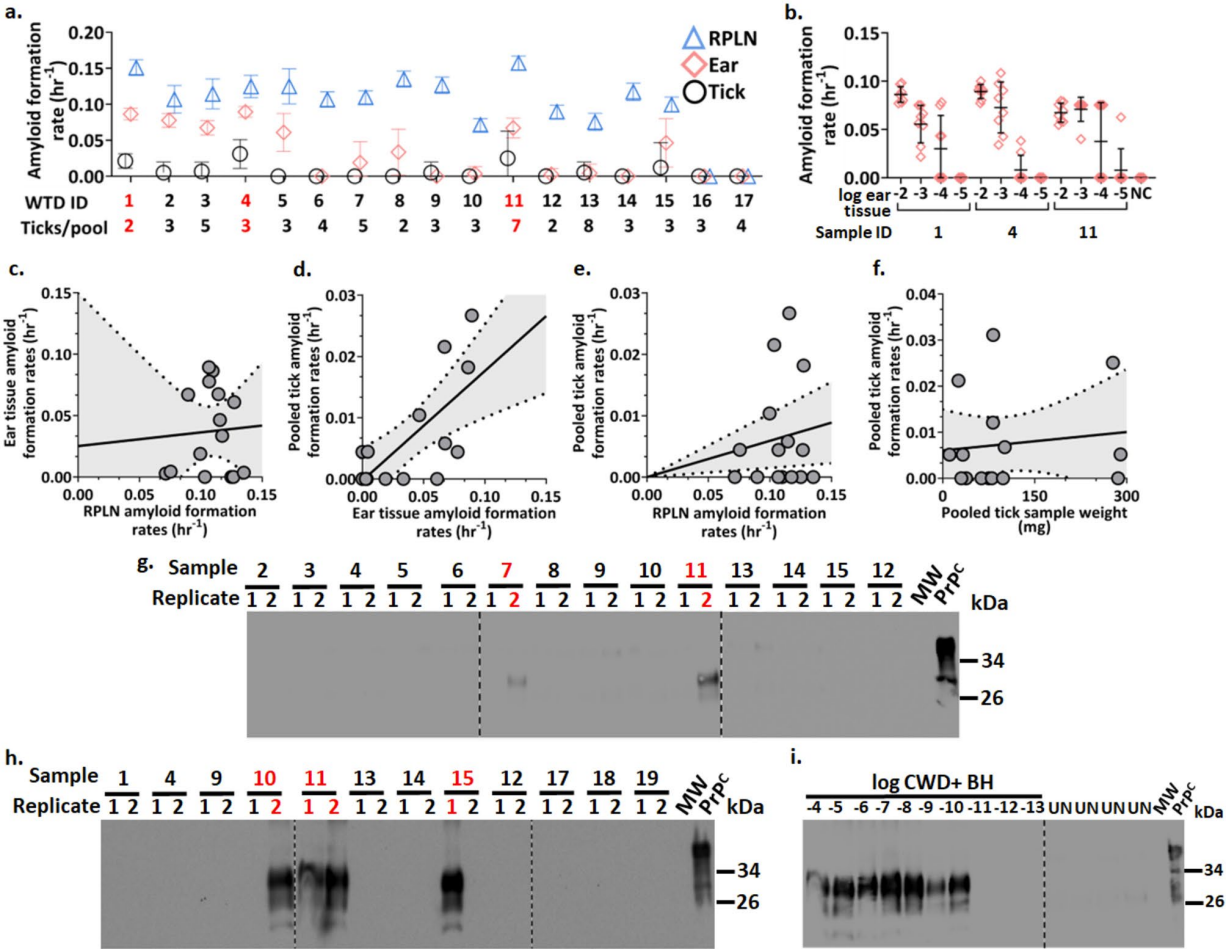
Presence of chronic wasting disease (CWD) prions (PrP^CWD^) in tissues and *Ixodes scapularis* from hunter-harvested white-tailed deer (WTD) assessed by two protein amplification assays. Comparisons of real-time quaking-induced conversation (RT-QuIC) amyloid formation rates (AFR) for (**a**) deer retropharyngeal lymph node (RPLN), ear tissue (pinna), and pooled tick samples from 15 CWD-positive (ID 1–15) and two of the 15 CWD-negative (ID 16,17) WTD (all other negative sample results are shown in Supplementary Table S2, Supplementary Figure S1 and S2) and (**b**) tenfold dilutions of ear tissue homogenates (ID 1,4,11). Samples are grouped by WTD IDs on the x-axis. Data points represent mean AFR ± standard deviation of 8 technical replicates. Negative controls (NC) represent the same sample types. (**c**–**f**) Scatterplots with fitted linear regression line with 95% confidence intervals comparing AFR relationships between (**c**) RPLN to ear samples, (**d**) tick to ear samples, (**e**) RPLN to tick samples, or (**f**) pooled tick samples with pooled tick sample weight in milligrams (mg). Each point represents the mean AFR of 8 technical replicates from WTD ID 1–15. (**g**–**i**) Western blot analysis of PMCA products to assess the presence of prion seeding activity for; (**g**) 14 pooled tick samples (WTD IDs 2–15) stored in RT-QuIC sample buffer evaluated in “a”; (**h**) 10% tick homogenates (ID 17–19 are NCs); (**i**) serial dilutions of a CWD-positive (CWD +) brain homogenate (BH) (PMCA positive control), and unseeded (UN) or cellular prion (PrP^C^) (PMCA NCs). (Original uncropped blots/gels are presented in Supplementary Figure S3) Samples analyzed in this figure were tested in duplicate and represent a third PMCA round. Numbers at the right of each panel represent molecular weight markers in kilodaltons (kDa). Red font indicates samples determined to contain PrP^CWD^ by either RT-QuIC or PMCA.

No false seeding activity was observed for tick or ear tissue samples collected from CWD-negative WTD. However, detection of PrP^CWD^ in these peripheral samples (ticks and ear tissue) was limited compared to detection in RPLN for each of the 15 CWD-positive WTD (Fig. 2a, Supplementary Table S2). Comparing AFR values among sample types, ear samples were positively correlated with wild-fed tick samples (*R*^2^ = 0.5, *t* = 3.62, *p*-value = 0.003, *N* = 15; Fig. 2d), suggesting that ticks may perform as well as ear tissues in detecting prions. However, RPLN AFRs were not correlated with seeding activity in either ear tissues (*R*^2^ = 0.15, *t* = 1.51, *p*-value = 0.155, *N* = 15; Fig. 2c) or tick samples (*R*^2^ = 0.02, *t* = 0.5, *p*-value = 0.628, *N* = 15; Fig. 2e), indicating that these peripheral samples had reduced sensitivity for detecting prions compared to the RPLN tissue samples in our study using RT-QuIC. Most of the ear samples showed positive seeding activity to only a 10^–2^ dilution using RT-QuIC, however those that demonstrated seeding activity out to a 10^–3^, 10^–4^, 10^–5^ dilution also appeared to be reflective of higher AFRs from the corresponding tick samples that we determined to be CWD-positive (Sample IDs 1 (7/8), 4 (7/8), 11 (3/8) (*p-*values 0.0722, 0.0015, 0.0182, respectively, using Dunnett’s Multiple Comparison Test) (Fig. 2a,b). These findings by RT-QuIC indicate a CWD prevalence of 20% (3/15) in *I. scapularis* based on this specific sample of CWD-positive WTD, and suggest that (i) circulation of PrP^CWD^ in peripheral tissues is associated with detectable levels of prionemia, which is consistent with previous evaluations of peripheral levels of PrP^CWD^ during presymptomatic and symptomatic stages of the disease^22,23^ and (ii) that ear tissue or attached and partially or fully engorged ticks may be a less sensitive sample source for CWD diagnostics compared to RPLN using RT-QuIC. Nevertheless, our data indicate that ticks may be considered as an *antemortem* detection method.

While it is possible that the variation observed in seeding activity from peripheral samples compared to RPLN may have been the result of differences in assay sensitivity for the different sample types, it may also be explained by other factors. The variation observed could have been influenced by the differences in total mass of tick per pooled tick sample; however, no correlation was found between higher or lower AFR values and higher or lower pooled tick samples mass (*R*^2^ = 0.02, *t* = 0.46, *p*-value = 0.66, *N* = 15; Fig. 2f). Additionally, differences in polymorphisms of the prion protein encoding gene (*PRNP*) can directly influence the rate of disease progression and distribution of PrP^CWD^ in WTD^24–27^. The WTD sample size in this study was not large enough to make inferences on how genotype may explain the variable distribution of seeding activity from tick or ear tissue in relation to activity from RPLN. However, genotyping results did show that all ticks from CWD-positive 96G/96S or 96S/96S animals were negative by RT-QuIC (Supplementary Table S1). This is in agreement with the previously suggested delayed accumulation of prions in peripheral tissues by animals harboring 96S alleles^26,27^.

Because the pooled tick samples collected from free-ranging CWD-positive WTD appeared to contain relatively low levels of PrP^CWD^ detectable by RT-QuIC, we employed an additional protein amplification assay, the protein misfolding cyclic amplification (PMCA) technique, to further assess the presence of PrP^CWD^ in these samples. This assay is akin to RT-QuIC, but uses brain extract from healthy rodents as a source of PrP substrate and relies on cycles of sonication rather than shaking^28^ for sensitive and specific detection of PrP^CWD^ (Fig. 2i). Relevant to this study, previous reports have shown that PMCA is able to amplify low levels of PrP^CWD^ from a wide variety of animal-derived and environmental samples^2,4–6,23,29–33^. From the 30 pooled tick samples collected from CWD-positive and negative WTD previously analyzed by RT-QuIC, PMCA-seeding activity was identified in four samples (Fig. 2g,h). Sample IDs 7, 10, and 15, which were negative based on RT-QuIC seeding activity, showed positive PMCA detection in 1/2 technical replicates. The fourth sample to test positive by PMCA, sample 11 (positive PMCA detection in 2/2 replicates) (Fig. 2g,h), was the only pooled tick sample that was positive based on both assays (Fig. 2a,g,h). Interestingly, this sample also demonstrated the highest seeding activity across all 15 WTD RPLN tested by RT-QuIC and had some of the most sensitive ear tissue seeding activity (Fig. 2a,b). These PMCA results demonstrate a CWD prevalence of 26.7% in *I. scapularis* based on this specific sample of CWD-positive WTD. However, if we consider where the detection of PrP^CWD^ agreed between the two amplification assays, the prevalence was only 6.7%.

**Figure 3:**
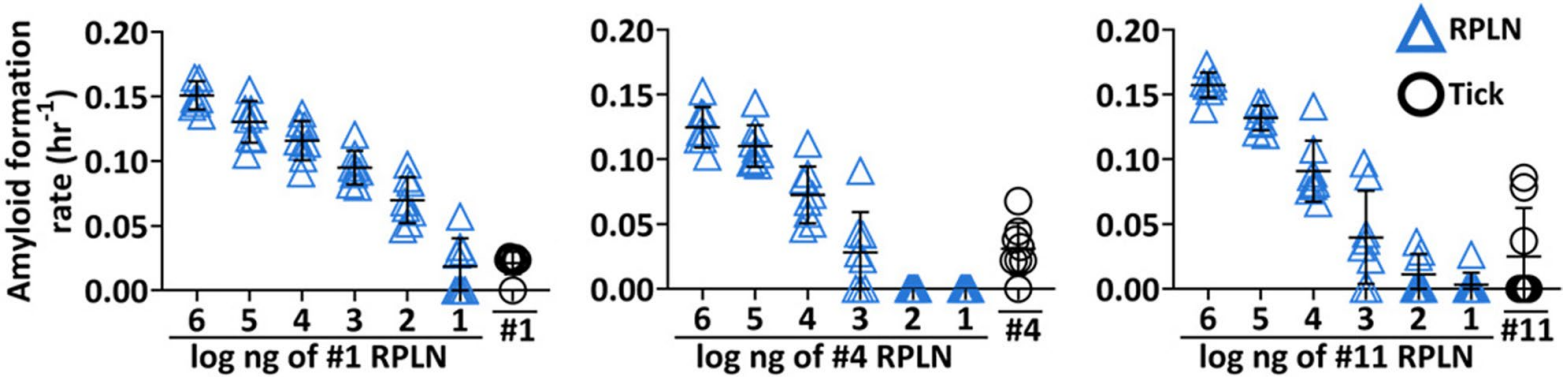
Comparison of chronic wasting disease (CWD) prion (PrP^CWD^) loads by *Ixodes scapularis* following infected blood meal with retropharyngeal lymph node (RPLN) from free-ranging white-tailed deer (WTD). Comparisons of relative PrP^CWD^ loads present in three separate pooled tick homogenates (ID numbers 1, 4, 11) from Fig. 2a, which tested CWD-positive by our parameters, against a tenfold dilution series of RPLN from corresponding WTD. Data points for 8 technical replicates are depicted for each sample ± standard deviation.

Because titers of PrP^CWD^ from a CWD-positive brain are similar to those found in CWD-positive RPLN^34^, we were able to first extrapolate predicted values for the mass of seeding material present in each RT-QuIC, CWD-positive pooled tick sample (Fig. 3). Then, based on the predicted mass of seeding material present and the previously reported minimum oral ID_50_ of CWD-positive brain^35^, we further estimated an ID_50_ for ticks from WTD samples 1, 4, and 11 to be 0.3, 42.4, and 6.9, respectively (Eqs. (1) and (3) in Data Analysis, Supplementary Table S3). These estimations suggest that a single *I. scapularis* tick taking a blood meal (i.e., fully, or partially engorged) from a CWD-positive WTD poses a risk to naïve individuals if consumed during social interactions.

## Discussion

Natural modes of indirect transmission of CWD among free-ranging cervids remain poorly examined and may perpetuate endemic increases and broad geographic spread of the disease^1^. The presence of PrP^CWD^ in blood may pose a risk for indirect transmission by way of hematophagous ectoparasites acting as mechanical vectors, as cervids can carry high tick infestations and exhibit allogrooming, a common tick defense strategy between conspecifics. However, the role of ticks as mechanical vectors of CWD remains unclear. Here, we demonstrate that a single adult *I. scapularis* found feeding on a CWD-infected WTD may contain approximately 0.3–42.4 ID_50_. These findings suggest that consumption of ticks by deer during bouts of allogrooming may facilitate oral exposure of PrP^CWD^ from ticks that consumed blood meals from CWD-infected deer. We demonstrate with artificial membrane feeding assays that *I. scapularis* has the capacity to ingest and excrete PrP^CWD^ and sensitivity and specificity of PrP^CWD^-exposed ticks with RT-QuIC validates the presence or absence of PrP^CWD^ in wild-fed ticks. Our results show that RT-QuIC seeding activities in wild-fed ticks were analogous to 10–1000 ng of CWD-infected RPLN from each animal (Fig. 3) and peripheral samples were less sensitive using RT-QuIC compared to RPLN (Fig. 2a). Using both RT-QuIC and PMCA, we showed seeding activity in 6 (4 by PMCA, 3 by RT-QuIC, 1 by both) of 15 pooled tick samples removed from wild CWD-infected white-tailed deer (Fig. 2a,g,h). These results indicate CWD prevalence ranging from 7 to 40% in *I. scapularis* that had fed on CWD-infected WTD and suggest that (i) the amount of PrP^CWD^ present in tick samples were near the detection threshold for each method and (ii) that when the amount of PrP^CWD^ present in a given sample is relatively low, multiple methods increase the chance of detection in ticks.

Although the ultra-sensitive capabilities of both RT-QuIC and PMCA are well established for detection of misfolded prions in blood from experimental and naturally occurring prion disease^8,21,36^ and lymph and skin tissues^37,38^, we observed variation between the two amplification assays in which pooled tick samples were determined CWD-positive (Fig. 2a,g,h). While both RT-QuIC and PMCA are clearly capable of detecting misfolded prions from blood samples, an understanding of how blood-engorged tick extracts behave in either assay is limited. Some sample types have highly sensitive and specific detection (e.g., RPLN), while others have lower sensitivity —often attributed to reaction inhibitors, depending on sample type and amplification assay, necessitating sample or assay optimization to improve sensitivity (e.g., saliva)^39^. Our optimization methods for both whole blood and blood-engorged ticks allowed for sensitive and specific detection of PrP^CWD^ from both sample types by RT-QuIC for the spiking experiments (Fig. 1a,h). However, whole blood contained within the engorged ticks may have influenced assay sensitivity, as whole blood components have been shown to inhibit detection of misfolded prion by RT-QuIC^40^ and PMCA^41^, which may explain the sensitivity differences between the two assays. The PMCA assay has been utilized to readily detect misfolded prions in blood seemingly without the need for extensive optimization to overcome inhibitors^23^ and was therefore used in this study to cross analyze the pooled engorged tick samples tested by RT-QuIC. Although we were able to detect the presence of PrP^CWD^ in pooled engorged tick samples by PMCA as well as RT-QuIC, the samples only detected a single sample in common. This relative inconsistency in sample detection between assays could be due to low circulating levels of misfolded prions, which is known to result in lower and inconsistent assay sensitivity^42^. Therefore, it is possible that the differences in sensitivity between RT-QuIC and PMCA observed in this study for detecting PrP^CWD^ from wild-fed tick samples may have been the result of low circulating levels of PrP^CWD^ in blood, hence low amounts of PrP^CWD^ present in tick samples. Additional research in this area could determine the comparative power of these two techniques for this specific sample type.

We fully recognize that the 10^–3^ mg/mL dilution used for the spiking blood meals for the membrane feeding assays is not typical of what is found in blood of early or late-stage CWD-infected deer and that based on results from McNulty et al.^21^, the relative concentration of prions in blood is likely ~ 3 orders of magnitude lower than a 10^–3^ mg/mL concentration of CWD-positive brain. However, considering results published by Shikiya et al.^12^, which found no uptake of prions by nymphal Rocky Mountain wood ticks (*Dermacentor andersoni*) following an animal challenge study, we felt it necessary to use such high concentrations of CWD-positive spiking material to demonstrate the capacity of *I. scapularis* to assimilate prions from a blood meal rather than demonstrate natural uptake under the most ideal controlled conditions. Future studies considering both—blood specimens spiked with more diluted prion titers (mimicking prion concentrations at different stages of the animal’s disease course) and blood from actual deer—would be important to consider when evaluating assay sensitivity between PMCA and RT-QuIC.

The observation of inconsistences of seeding activity by ear or tick samples compared to seeding activity of RPLN from the 15 CWD-positive WTD may be the result of differences in the stage of CWD disease progression across the individuals sampled, as variation in disease stage could influence PrP^CWD^ distribution^43^. Although we cannot confirm disease stage for any of the sampled WTD included in this study, this sample set of WTD was not randomly selected, but rather based on those selectively harvested by hunters, making it unlikely that any of the sampled individuals were in the end-stages of CWD. Hence, there is an expectation that the ear tissue and pooled tick samples would contain relatively low PrP^CWD^ levels, given lower blood flow to ear tissue, the small sample volumes taken up by adult ticks, and that it is unlikely that a deer in the terminal stage of CWD would have been targeted for hunter harvest. Although PMCA and RT-QuIC are capable of detection of misfolded prions at femtogram levels—similar to the lower detection limit of bioassay^44,45^—the inconsistencies in sensitivity between the two assays for our sample set may have resulted from having samples that contain PrP^CWD^ levels below detection thresholds for even these ultra-sensitive amplification assays. This possible detection limit may explain why WTD 11 was the only deer with a pooled tick sample that overlapped in positivity between the two assays (Fig. 2a,g,h). The elevated RPLN AFRs and more sensitive seeding of ear tissue from sample 11 compared to all other RPLN and ear tissue samples suggests that this individual had higher circulating amounts of PrP^CWD^ levels that were at or above the detection threshold between the two assays (Fig. 2a,b). Future work aimed at better understanding how the range of PrP^CWD^ levels that ticks may harbor affects assay sensitivity and detection rates could incorporate a broader sample set that includes samples from deer that are in the end-stages of CWD. Additionally, this study only investigated ticks concentrated around the head and neck of deer. It is possible that ticks collected from other anatomical regions lacking a common vasculature with the head may exhibit different PrP^CWD^ detectability and prevalence. Further studies investigating engorged ticks across the entire anatomy of WTD are warranted.

While variation of seeding activity observed in pooled tick and ear tissue samples across the individual free-ranging WTD from this study may demonstrate limits of detection for both PMCA and RT-QuIC, the naturally occurring PrP^CWD^ loads from ticks and ear tissue collected from the 15 CWD-positive WTD may also be influenced by *PRNP* genotype^27^. Although the small sample size of CWD-positive animals in this study limited our ability to assess how genotype alters variation in seeding activities, we found that RT-QuIC tested pooled tick samples from CWD-positive 96G/96S or 96S/96S animals were negative and positive pooled tick samples came from CWD-positive 96G/96G. The four positive PCMA results for the same pooled tick samples showed detection of PrP^CWD^ from one CWD-positive 96G/96S, and three CWD-positive 96G/96G (See Supplementary Table S1 for genotype results and more information regarding genotypes). An additional consideration is that CWD strain variation may be another plausible explanation for the distribution in seeding activity across the sample types examined in this study, as peripheral and neural PrP^CWD^ distribution can differ across recognized CWD strains^46^.

Indirect transmission routes of CWD likely play an important role in CWD disease dynamics^1^ and are quite possibly a combination of various modes of exposure that may include consumption or inhalation of contaminated soil^47,48^, consumption of contaminated plant matter^4^, or mucosal contact with contaminated fomites or other environmental materials^49^. Few studies have utilized RT-QuIC to evaluate the involvement of other species in the ecological community that may influence CWD exposure or transmission. We have identified a potential mechanical vector of CWD not previously evaluated for WTD, with implications for host behavior that may influence CWD exposure events. We recognize that experimentally-determined oral ID_50_ of our tick samples may vary significantly from our estimates, as it is quite evident different prion loads were present in each animal based on the RPLN dilution series (Fig. 3), and the prior study^35^ used to estimate the ID_50_ utilized a pool of infectious material generated from laboratory-infected late-stage animals. As such, these can be considered preliminary estimates for ID_50_ in ticks; our sample size is small and experimentation in mouse models would establish a true ID_50_. Nevertheless, our findings suggest that infection relevant loads of seeding material are indeed present in individual ticks, likely within an order of magnitude of 1 ID_50_. These findings and implications may prove useful for CWD research and adaptive management efforts moving forward as we advance our understanding of ecologically relevant drivers of CWD dynamics. Future studies could clarify the prevalence of CWD in ticks for a single deer and explore the potential importance of the relationship between tick CWD prevalence and where on the host’s body the tick attaches. Although the detection rate varied depending on the seeded amplification assay being used, this was not surprising as it is well known that biological and environmental specimens may carry components affecting the PrP^C^–PrP^CWD^ conversion process. Future studies could determine whether sample pre-treatments or modifications in the RT-QuIC or PMCA processes increase prion detection ratio in ticks and other parasites. Importantly, future research including bioassays, evaluating larger samples sizes of *I. scapularis* and other tick species collected from WTD, and additional wild cervid species across different regions of North America would expand our understanding of the role that blood obligate ectoparasites and associated biogeographical factors may have on CWD dynamics. For example, land use change and shifts in regional climate regimes may result in higher tick infestations on cervids and contemporary range expansion for different tick species^50^, potentially increasing the likelihood of this type of exposure event among WTD and other cervids. Additionally, behavioral studies evaluating cervid allogrooming frequency, rate, and preferences for allogrooming across different cervid species could shed light on links between host behavior, disease status and conspecific exposure risk. For example, do end-stage CWD-infected cervids accumulate higher tick burdens (suggested in^51^) through altered grooming or habitat selection behaviors? Do healthy individuals continue to groom infected conspecifics or do they avoid allogrooming interactions with visibly sick individuals? Future research efforts could help elucidate the impact of ticks and grooming behaviors on transmission of CWD in free-ranging cervids.

## Methods

### Membrane fabrication and feeding chamber assembly

The tick artificial membrane-feeding method used was based on Oliver et al.^20^, with some modifications. Briefly, silicone membranes were produced by infiltrating 50% rayon and 50% cellulose microscope lens paper (Matin) with a two-component liquid silicone rubber with a shore hardness of 00–50 (Ecoflex Supersoft 0050, Smooth-On, PA, USA), mixed 1:1 and thinned by adding 1.5 mL hexane into 10 mL silicone mixture (Fig. 1e). The lens cleaning paper was taped to a flat, plastic wrap-lined surface and the silicone mixture scraped over the lens cleaning paper to obtain a membrane with a thickness of ~ 70–100 µm. Membrane thickness was determined by measuring 4–6 points on each cured membrane with a micrometer. Membranes were allowed to cure overnight before feeding chamber attachment using a 1:1 mixture of a two-part silicone glue with shore A hardness of 30 (Mold Star 30, Smooth-On) as previously described, resulting in 4–6 feeding chambers per cured membrane sheet (Fig. 1i). Assembled feeding chambers were cured overnight, trimmed with a scalpel, and leak-tested by adding 5 mL of sterile water to each chamber for at least 1 h, discarding any leaky membranes. To ensure feeding chambers remained upright and membranes were submerged into blood-containing wells, plastic graduated cylinder bumpers were placed around feeding units and glued into place with silicone glue (Gorilla Glue) (Fig. 1f,g).

### Tick housing and feeding

Pathogen free *I. scapularis* male and female adults were acquired from the Oklahoma State Tick Rearing facility, Stillwater, Oklahoma, U.S.A., and housed in a humidity chamber (Durabilt, 64-quart clear storage tote with lid) at 24 °C with 97–99% relative humidity (Durac, hygrometer). Humidity was maintained by placing lidless, smaller separate plastic containers containing a saturated potassium sulfate solution (~ 0.5 kg K_2_SO_4_ (potassium sulfate) submerged in ~ 500 mL of sterile deionized water) within the larger humidity chamber. Ticks were acclimated to the humidity chamber for ~ 5 h prior to starting artificial membrane feeding. Sterile, mechanically defibrinated bovine blood (Hemostat Laboratories, Dixon, CA) was used. Three mL of blood supplemented with 4.5 µL of 3 mM ATP was prewarmed to 37 °C, then added to each well of a six-well plate to stimulate feeding^52^. In total 10–14 female and 5–7 male *I. scapularis* were placed in each feeding chamber enclosed using a fine synthetic mesh fabric (Anteer Crystal Organza, China), fixed in place with a tight rubber band. If available from previous feedings, 10–15 granules of tick frass were added to each feeding chamber as a feeding stimulant. Feeding chambers were positioned in direct contact with the blood without trapping air bubbles between the membrane and the blood, submerging membranes by at least 2 cm. Each plate was placed afloat in a 37 °C water bath in a room that provided a 16:8 h photoperiod. Every 24 h, blood was replaced by placing 3 mL of prewarmed bovine blood as described above in a new six-well plate. To remove the build-up of blood residue from the previous day’s feeding, the outer surface of each feeding chamber and membrane that were in contact with the blood were vigorously rinsed with sterile 1X phosphate-buffered saline (PBS) using a pipette.

### Exposure of *I. scapularis* to CWD by membrane feeding

To determine tick uptake of PrP^CWD^ from inoculated blood meals, we inoculated 2.970 mL of defibrinated bovine blood with a 30 µL of a 10% (w/v) CWD-positive WTD brain homogenate (from the obex region; sourced from Wisconsin Department of Natural Resources (WDNR) tagged WTD #5219) prepared in 1X PBS to achieve a final concentration of 10^–3^ mg/mL of CWD-positive brain for the CWD-positive treatment group. For the negative control treatment group, 2.970 mL defibrinated blood was treated with 30 µL of 1X PBS. Separate six-well plates were used for each treatment group and each six-well plate with feeding chambers attached were placed afloat in a 37 °C water bath that was housed within a humidity chamber (24 °C with 97–99% relative humidity). Every 24 h, feeding unit plates were replaced and refilled with fresh blood for each treatment group. The outer surfaces of the feeding chamber and membranes were washed as detailed above, washing the negative control feeding units first and the CWD-positive treatment feeding chambers last to prevent any possibility for cross contamination. Ticks were allowed to feed to repletion for ~ 9–14 days, during which frass was also collected from each treatment group. Engorged, detached ticks and frass were stored at − 20 °C prior to preparation for RT-QuIC or PMCA analyses.

### Collection of ear tissue and ticks attached to hunter harvested, wild white-tailed deer

Through cooperation with the WIDNR CWD processing center, heads of voluntarily submitted hunter-harvested WTD from several Wisconsin counties were manually examined for ticks from October 2021 to December 2021. Examinations generally consisted of combing fingers through pelage for attached ticks on each WTD head while wearing nitrile gloves. When detected, attached ticks were removed, typically found on the outer ear, brow, chin, cheek, snout, or neck regions. Each head was contained within a leak-proof disposable plastic bag and tagged with a WIDNR barcode for identification and linking to metadata for CWD testing. To prevent cross contamination of each deer head being examined, heads were kept within their respective bags, gloves were changed between each head examination, and a new sterile scalpel was used for removal of the entire left ear of each head. Each ear sample was placed in a sterile Whirl–Pak bag (Nasco, 48,137), and any ticks collected from a given head were collectively placed in sterile 1.5 mL snap-cap centrifuge tubes. All samples were labeled with the respective barcodes for each head examined, and the number and species of tick collected from CWD-positive WTD were cataloged. Tissue and tick samples were stored at − 20 °C prior to preparation for use in the RT-QuIC or PMCA assays.

### Tick, blood, and frass homogenate preparations for RT-QuIC

To prepare tick homogenates, a mix of partially and fully engorged *I. scapularis* female ticks equaling a total weight of ~ 200 mg (unless stated otherwise, equivalent to ~ 142 µL of blood meal), were placed in a ring-sealed 1.5 mL centrifuge tube with ~ 30–40 0.7 mm zirconia beads (BioSpec) with 1 mL of PBS and processed at room temperature in a bead mill homogenizer (Fisherbrand Bead Mill 24) on the highest setting (setting six) for 3 min. The mass of each tick sample collected from hunter-submitted deer heads varied and was therefore prepared using volumes of 1X PBS to result in a 10% (w/v) homogenate. Next, tick homogenates were mixed with chitinase (1 mg/ mL final concentration) (Sigma-Aldrich Cat. # C824) and allowed to digest in a thermomixer (1,400 rpm, 24 h, 45 °C; Eppendorf ThermoMixer F1.5). Following digestion, Lipase AY30 (100 µg/mL final concentration; Acros Organics) was added, and homogenates were thermomixed for 1 h (37 °C, 1400 rpm; Eppendorf ThermoMixer F1.5), followed by centrifugation (25 °C, 15 min, 16,000×*g*). Supernatants were collected, centrifuged again to ensure sample clarification (25 °C, 15 min, 16,000×*g*), mixed 1:1 with 23.1 mM sodium phosphotungstate hydrate (Na-PTA) (Sigma-Aldrich, Cat. # 496,626), incubated without agitation for 16 h at 4 °C. Samples were then centrifuged (4 °C, 30 min, 5000×*g*), pellets were retained and washed with a 1:1 solution of 18 MΩ H_2_O and 23.1 mM sodium phosphotungstate (Na-PTA) followed by centrifugation (4 °C, 30 min, 5000×*g*) and aspiration of the wash solution. Pellets were resuspended in 30 µL of RT-QuIC sample buffer (0.1% SDS in 1X PBS and N2 supplement (Gibco, 17,502,048)) using sonication (1 min, amplitude 36; Qsonica Q-700), and 2 µL was used to seed each reaction well of the 96 well-plate for the RT-QuIC assay. Eight technical replicates were used per biological replicate, unless stated otherwise.

For spiking experiments using tick homogenates for RT-QuIC optimization, the initial volume of the spiked sample consisted of 450 µL of negative control tick homogenate (as prepared above), 450 µL chitinase (1 mg/ mL final concentration), and 100 µL of a 10^–3^ dilution of either CWD-positive or CWD-negative WTD brain. Then the steps outlined above for preparing tick homogenates were performed, with Na-PTA pellets resuspended in 100 µL and ten-fold dilutions were prepared from the 10^–3^ spiked sample. Two µL of each dilution were used to seed each reaction well for eight technical replicates. Spiking experiments used to demonstrate recovery of PrP^CWD^ from defibrinated bovine blood were carried out using 200 µL of blood combined with 1 mL PBS, followed by the homogenization step with an additional 1 mL of PBS added. The initial volume of the spiked blood samples consisted of 450 µL blood homogenate, 450 µL PBS, and 100 µL 10^–3^ CWD-positive of CWD-negative WTD brain homogenate dilution. Samples were incubated in a thermomixer for 16 h (45 °C, 1400 rpm), then 1.5 µL of Lipase AY30 (100 µg/mL final concentration; Acros Organics) was added and the samples homogenized with Na-PTA pellets resuspended in 100 µL of RT-QuIC sample buffer and tenfold dilutions were prepared from the 10^–3^ spiked sample, using 2 µL of each dilution to seed each reaction well for eight technical replicates per biological replicate. For RT-QuIC analysis of tick frass from feeding experiments, 70 mg of frass from each treatment group was added to 1 mL of 1X PBS, followed by homogenization. Lipase AY30 (100 µg/mL final concentration) was added, and samples were incubated in a thermomixer for 1 h (37 °C, 1400 rpm), centrifuged (16,000×*g*, 15 min, 25 °C), and supernatants were collected. Next, 500 µL of 1X PBS was added to 500 µL of the supernatant, then mixed 1:1 with 23.1 mM sodium phosphotungstate followed by incubated without agitation for 16 h at 4 °C, Na-PTA pellets were centrifuged, washed, and centrifuged again, then resuspended in 30 µL with sonication. Then, 2 µL of each sample was used to seed each reaction well for 8 technical replicates.

### Tissue homogenate preparation for RT-QuIC

Ear tissue was prepared as described in Burgener et al.^53^. Briefly, 100 mg of ear tissue collected from the central area of the pinna was placed in a digestion solution (1X PBS, 2 mM CaCl_2_ (Dot Scientific DSC20010-1000), and collagenase A (2.5 mg/mL final concentration) (Sigma-Aldrich 10,103,586,001)) were homogenized with a bead beater (1 min, 4 m/s; Fisherbrand Bead Mill 24) and 0.7 mm diameter zirconia beads (BioSpec). These samples were then further processed with a thermomixer (1400 rpm, 24 h, 45 °C; Eppendorf ThermoMixer F1.5), after which they were centrifuged (2 min, 3000×*g*), and the supernatants retained. The supernatants were centrifuged again (3 min, 3000×*g*) to remove any small particulate matter, aliquoted, and frozen at − 20 °C until use for RT-QuIC analysis.

### Real-time quaking-induced conversion assay

The RT-QuIC in vitro protein amplification assay was performed as described by Metrick et al.^54^ with minor modifications. Briefly, 2 µL of sample extracts were added to a given well of a 96-well format optical-bottom black microplate (Fisher), each already containing 98 µL of RT-QuIC reaction mixture (0.1 mg∙mL^−1^ 90–231 recombinant hamster prion protein (produced as previously described by Orru et al.^55^, 300 mM sodium iodide, 20 mM sodium phosphate, 1.0 mM ethylenediaminetetraacetic acid, and 10 µM thioflavin T). Microplate-compatible spectrophotometers capable of heating, shaking, and fluorescence monitoring (BMG FLUOstar, Cary, NC) were used with the following instrument settings: 50 °C for spiked samples (unless described otherwise) double orbital pattern shaking at 700 rpm with 60-s shake/60-s rest cycles, fluorescent scans (λ_excitation_ = 448 nm, λ_emission_ = 482 nm) every 15 min, at a gain of 1600, and a total run time of 48 h.

### CWD status by ELISA

Retropharyngeal lymph nodes collected from hunter-harvested deer were tested by enzyme-linked immunosorbent assay (ELISA) using the standard protocol approved by the U.S. Department of Agriculture (USDA) at the Wisconsin Veterinary Diagnostic Center, Madison, Wisconsin, U.S.A. The ELISA assay was conducted using a commercial Transmissible Spongiform Encephalopathy Antigen Test kit (Bio-Rad, Catalogue# 12,004,413) (bovine obex or mule deer/WTD/elk RPLN and obex), following manufacturer’s instruction. Identification of the presence of CWD is based on an optical density (OD) value that is equal to or greater than the USDA cut-off value (0.035).

### Protein misfolding cyclic amplification

The PMCA substrate was generated from a pool of brains from Tg(CerPrP)1536^+/+^^56^ mice as described in Morales et al.^28^. PMCA substrate was supplemented with digitonin (Invitrogen, Carlsbad, CA, USA) and EDTA (Promega, Madison, WI, USA) at final concentrations of 0.025% and 6 mM, respectively. Aliquots of 90 µL of PMCA substrate were transferred in 0.2 mL PCR tubes strips (Eppendorf, Enfield, CT, USA) containing PTFE beads (Engineering Laboratories, Inc., Oakland, NJ, USA) and mixed with 10 µL of tick-derived samples. Tick samples used for PMCA were prepared in either RT-QuIC sample buffer or as a clarified homogenate following treatment with chitinase, Lypase AY30, and centrifugation to further clarify the sample prior to adding Na-PTA as described above in the “Tick, blood, and frass homogenate preparations for RT-QuIC” section above. The PMCA reactions were submitted to a first round of 144 cycles of incubation/sonication. The resulting PMCA products (10 µL) were mixed with fresh PMCA substrate supplemented (90 µL) and subjected to two additional PMCA rounds of 96 cycles each. Each PMCA cycle consisted of 29 min., and 40 s of incubation, and 20 s of sonication at 37 °C. Each PMCA reaction set included PMCA reactions spiked with serial dilutions of CWD-positive brain (10 µL) of known PMCA activity and 4 unseeded reactions as negative controls. PMCA products were mixed with proteinase K (PK, Sigma-Aldrich, Saint Louis, MO, USA) at final concentration of 100 µg/mL and incubated at 37 °C for 90 min with shaking. The PK catalytic activity was stopped by adding NuPAGE LDS sample buffer (Invitrogen, Carlsbad, CA, USA) at final concentration of 1X and heated at 90 °C for 10 min. PK-treated PMCA products were visualized by western blot using the Bar-224 antibody (Bertin Corp, Rockville, MD, USA) at 1:10,000 dilution. PMCA manipulators were blinded to the identity of the samples.

### PRNP analysis

Genomic DNA was extracted from ~ 100 mg of ear tissue from 17 out of 30 WTD included in this study using methods outlined in Green and Sambrook, 2012 for phenol–chloroform extraction and ethanol precipitation^57^. An approximately 750 bp *PRNP* gene sequence was amplified by conventional PCR and sequenced at the University of Wisconsin Biotechnology Center (Madison, Wisconsin, U.S.A) using primer sequences developed by O’Rourke et al*.*^24^. PCR sequences were then aligned and evaluated using Unipro UGENE software version 42.0 (www.ugene.net). Specific single nucleotide polymorphisms at position 95 (glutamine [Q] or histidine [H]), 96 (glycine [G] or serine [S]) were identified and recorded. Although an updated set of primers which accounts for rare *PRNP* alleles was recently identified^58,59^ following the initial submission of our findings, primers used for this study have demonstrated consistent utility for the past decade^25,60^.

### Data analysis

Data were analyzed and visualized using Jmp Pro 15 (SAS Institute, Cary, NC) and Prism 8 (GraphPad, San Diego, CA). Thresholds used to determine AFRs were calculated by adding twenty times the standard deviation of the relative fluorescence unit (RFU) values from cycles 3–14 to the mean of RFU values from cycles 3–14 to account for baseline variation amongst samples and to apply a rigorous standard for distinguishing true positive samples from true negatives.

We first evaluated if we could recover and detect PrP^CWD^ from spiked tick homogenates or spiked blood as compared to the source material (CWD-positive brain tissue), and if the recovery rates differed by sample type. We used a two-way (factorial) analysis of variance (ANOVA) to compare AFR values among sample types (CWD-positive brain, spiked-blood or spiked tick homogenates). We included an interaction between sample type and sample dilution, to assess if detection/recovery in different sample types was sensitive to the sample concentration across the tenfold dilution series.

After this proof of concept, we then evaluated whether ticks experimentally fed blood inoculated with CWD-positive BH could ingest and excrete prions. We used a two-way (factorial) ANOVA to assess differences in AFR values based on sample type (CWD-positive brain tissue, ticks fed prion-spiked blood, or frass from the experimentally-fed ticks) and the interaction between sample type and sample dilution across the tenfold dilution series.

To analyze whether PrP^CWD^ was detectable in engorged ticks collected from free-ranging CWD-positive deer, we compared results generated by two protein amplification assays, RT-QuIC and PMCA. Pooled tick samples amplified by PMCA that demonstrated bands between 34 and 26 kDa were interpreted as being positive for having PrP^CWD^ present^61^. Pooled tick samples analyzed by RT-QuIC were considered positive if a sample had at least 3 out of 8 technical replicates with seeding activity and also by statistical analysis, using Dunnett’s multiple comparison test of AFR values to distinguish which pooled tick samples were significantly different from the negative control pooled tick samples collected from free-ranging CWD-negative WTD heads.

Since CWD testing in free-ranging cervids currently relies upon invasive sampling of RPLN, we explored if CWD status could be assessed through more readily accessible tissues (such as ear tissue or ectoparasites) which could provide support for performing less invasive *antemortem* CWD testing. Therefore, we collected RT-QuIC average AFR values for three sample types (lymph node, ear, or pooled tick samples) from individual deer to evaluate if AFR values were correlated (i.e., do deer with high AFR values in lymph nodes have higher AFR values in ear or tick samples than deer with lower AFR values?). Using linear regressions, we explored relationships between average AFRs for the three sample types collected from the CWD-positive deer in our sample set.

We then calculated if prion concentrations detected in ticks from free-ranging deer had the potential to be infectious, based on estimates of the amount of prion seeding material in our samples relative to an experimentally-determined ID_50_ for an equivalent amount of prion seeding material in brain^35^. To estimate a predicted per-tick ID_50_ (ID_50P_) we need to know the ng of predicted seeding material per 1 mg of tick for a pooled sample (*S*), the average mass (mg) for a single tick from a pooled sample (*m*) from WTD ID 1, 4, and 11, and the actual ID_50_ of an equivalent mass of CWD-positive brain (300 ng = ID_50A_):

1 $${ID}_{50P}=\left(S\times m\right)\div {ID}_{50A}$$

Titers of PrP^CWD^ from a CWD-positive brain are similar to those found in CWD-positive RPLN^34^, and a recent study has described a minimum mass of 300 ng of CWD-positive brain derived from a pool of 6 CWD-positive deer to be an effective oral ID_50_ for WTD^35^. As such, to calculate *S*, we first modeled, using a sum of exponential functions, the AFR values generated for the tenfold dilution series using mass of RPLN tissue per 2 µL (the amount of sample used to seed each well) for each of the RPLN samples (1, 4, and 11) as the explanatory variable. Thus, our global model was:

2 $${AFR}_{i}=a+b\times \mathrm{exp}\left(-d\times {ng}_{i}\right)+c\times \mathrm{exp}\left(-f\times {ng}_{i}\right),$$

where ${AFR}_{i}$ is the observed AFR for the *i*th observation, *a, b, c, d* and *f* are parameters that are estimated and ${ng}_{i}$ is the mass of sample for the *i*th observation. Parameters for each model were estimated using a least squares loss function within the Nonlinear Fit Curve Personality of Jmp Pro 15 (SAS Institute, Cary, NC). For each dataset we examined a suite of 4 models, which were based on the global model described above with either 2, 3, 4, or 5 parameters. We used the Akaike information criterion (AIC_c_) corrected for small sample size, to select which model from this suite of models provided the most parsimonious fit for each of the three RPLN data sets. (see Supplementary Table S4 and S5 for AIC_c_ values and associated suite of models examined)^62^. Based on AIC_c_ values, the global 5-parameter model was chosen for data for sample #1 and the 4-parameter model best fit data for samples #4 and 11. The fitted models were then used as calibration curves to predict the relative amount of seeding material present in 2 µL of each pooled tick sample (For full model equations see Supplementary Table S4). Therefore, if *ng*_*p*_ is the predicted mass of seeding material in a 30 µL volume (the total volume of prepared pooled tick sample in RT-QuIC sample buffer, see Supplemental Material Methods section), and *M*_*t*_ is the total mass of (mg) of a pooled tick sample, then *S*, the ng of seeding material per 1 mg of tick for a pooled sample, is estimated as:

3 $$S=\left({ng}_{P}\right)\div \left({M}_{t}\right)$$

### Ethics approval

All animal manipulations were approved by the Animal Welfare Committee (AWC) at The University of Texas Health Science Center at Houston. Protocol number AWC-20–0065. All procedures were conducted following Federal and University guidelines. All methods are in accordance with ARRIVE guidelines. Mice were bred in approved facilities and euthanized by CO_2_ inhalation. The experiments listed in this manuscript did not involve animal experimentations and only tissues from euthanized mice were used as reagents for the PMCA reactions.

## Supplementary Information

Supplementary Information.

## Data Availability

The datasets generated and/or analyzed during the current study^63^ are available at www.sciencebase.gov using https://doi.org/10.5066/P9CAMSWN.
