# Supplementary material for: Ticks harbor and excrete chronic wasting disease prions
Source: Sci Rep. 2023 May 15;13:7838. doi: 10.1038/s41598-023-34308-3 (PMC10185559; doi:10.1038/s41598-023-34308-3)
Supplement: Supplementary file 1 — Supplementary Information. [file 41598_2023_34308_MOESM1_ESM.docx]

**SUPPLEMENTARY MATERIAL**

**SUPPLEMENTARY TABLES AND FIGURES**

**Supplementary Table S1.** Summary of genotype, age, sex, Wisconsin county harvested from, and chronic wasting disease (CWD) status by retropharyngeal lymph nodes (RPLN), ear, or pooled tick samples using real-time quaking-induced conversion (RT-QuIC) for 15 CWD-positive and two (out of 15) CWD-negative white-tailed deer (WTD) included in this study. Polymorphisms in prion protein gene (*PRNP*) can directly influence the rate of disease progression and distribution of CWD prions (PrP^CWD^) in WTD [23-25]. White-tailed deer (WTD) homozygous for glycine at the 96th allele of the *PRNP* gene (96GG) differ in duration of disease progression compared to those that are hetero- or homozygous for serine (96GS/96SS) [25,60,61] and WTD expressing amino acid histidine (H) at the 95th allele have limited peripheral accumulation of PrP^CWD^ compared to those expressing glutamine (Q) (H95/Q95) [45].

| **WTD ID** | **Genotype** | **Age** | **Sex** | **Hunter harvest county** | **CWD status by RT-QuIC RPLN/ear/tick** |
| --- | --- | --- | --- | --- | --- |
| 1 | GG | 3 | F | Richland | +/+/+ |
| 2 | GG | 1 | M | Dane | +/+/- |
| 3 | GG | 3 | F | Iowa | +/+/- |
| 4 | GG | 4 | M | Dunn | +/+/+ |
| 5 | GS | 3 | M | Columbia | +/+/- |
| 6 | GG | 4 | F | Iowa | +/-/- |
| 7 | GG | 1 | M | Dane | +/-/- |
| 8 | GG | 3 | M | Iowa | +/+/- |
| 9 | GG | 3 | F | Iowa | +/-/- |
| 10 | GG | 3 | M | Dane | +/-/- |
| 11 | GG | 2 | M | Sauk | +/+/+ |
| 12 | SS | 3 | M | Columbia | +/-/- |
| 13 | GG | 1 | M | Iowa | +/-/- |
| 14 | GS | 1 | F | Dane | +/-/- |
| 15 | GS | 2 | M | Marquette | +/+/- |
| 16 | GG | 2 | F | Monroe | -/-/- |
| 17 | GS | 3 | F | Richland | -/-/- |

**Supplementary Table S2.** Summary of results for pooled tick samples tested by real-time quaking-induced conversion (RT-QuIC) and protein misfolding cyclic amplification (PMCA) assays. NA indicates negative control samples that were evaluated by one assay but not the other.

| **Sample ID** | **Deer CWD status** | **Tick PMCA results** | | **Tick RT-QuIC results** | |
| --- | --- | --- | --- | --- | --- |
|  |  | **+/-** | **Technical replicates with seeding activity** | **+/-** | **Technical replicates with seeding activity** |
| **1** | **+** | **-** | **0/2** | **+** | **7/8** |
| **2** | **+** | **-** | **0/2** | **-** | **1/8** |
| **3** | **+** | **-** | **0/2** | **-** | **1/8** |
| **4** | **+** | **-** | **0/2** | **+** | **7/8** |
| **5** | **+** | **-** | **0/2** | **-** | **0/8** |
| **6** | **+** | **-** | **0/2** | **-** | **0/8** |
| **7** | **+** | **+** | **1/2** | **-** | **0/8** |
| **8** | **+** | **-** | **0/2** | **-** | **0/8** |
| **9** | **+** | **-** | **0/2** | **-** | **1/8** |
| **10** | **+** | **+** | **1/2** | **-** | **0/8** |
| **11** | **+** | **+** | **2/2** | **+** | **3/8** |
| **12** | **+** | **-** | **0/2** | **-** | **0/8** |
| **13** | **+** | **-** | **0/2** | **-** | **1/8** |
| **14** | **+** | **-** | **0/2** | **-** | **0/8** |
| **15** | **+** | **+** | **1/2** | **-** | **1/8** |
| **16** | **-** | **NA** | **NA** | **-** | **0/8** |
| **17** | **-** | **-** | **0/2** | **-** | **0/8** |
| **18** | **-** | **-** | **0/2** | **NA** | **NA** |
| **19** | **-** | **-** | **0/2** | **NA** | **NA** |
| **20** | **-** | **-** | **0/1** | **-** | **0/8** |
| **21** | **-** | **-** | **0/1** | **-** | **0/8** |
| **22** | **-** | **-** | **0/1** | **-** | **0/8** |
| **23** | **-** | **-** | **0/1** | **-** | **0/8** |
| **24** | **-** | **-** | **0/1** | **-** | **0/8** |
| **25** | **-** | **-** | **0/1** | **-** | **0/8** |
| **26** | **-** | **-** | **0/1** | **-** | **0/8** |
| **27** | **-** | **-** | **0/1** | **-** | **0/8** |
| **28** | **-** | **-** | **0/1** | **-** | **0/8** |
| **29** | **-** | **-** | **0/1** | **-** | **0/8** |
| **30** | **-** | **-** | **0/1** | **-** | **0/8** |
| **31** | **-** | **-** | **0/1** | **-** | **0/8** |
| **32** | **-** | **-** | **0/1** | **-** | **0/8** |

**Supplementary Table S3.** Derived values and the associated estimated individual tick ID_50_ based on the amyloid formation rate (AFR) prediction model with the lowest Akaike information criterion corrected (AIC*c*) value for 10-fold dilutions of retropharyngeal lymph node tissue from white-tailed deer (WTD) #1, 4, and 11. Parameter names in bold-italics indicate variable names for equations in text.

| **Estimated Tick ID_50_** | | | | | | | | |
| --- | --- | --- | --- | --- | --- | --- | --- | --- |
| Sample # | Predicted ng of seed in 2 µl | Predicted ng of seed per µl | Number of ticks in sample | Predicted ng of seed per  30 µl – (***ng_P_***) | Tick sample mass (mg) total – (***M_t_***) | ng of seed per mg of  tick – (***S***) | Average mass (mg) for a single tick – (***m***) | Predicted ID_50_ per tick –(***ID_50P_***) |
| 1 | 13.1 | 6.55 | 2 | 196.5 | 25.6 | 7.68 | 12.8 | 0.3 |
| 4 | 2545 | 1272.5 | 3 | 38,175 | 82.2 | 464.4 | 27.4 | 42.4 |
| 11 | 950 | 475 | 7 | 14,250 | 277.5 | 51.35 | 39.64 | 6.9 |

**Supplementary Table S4.** The model descriptions and Akaike information criterion corrected (AIC*c*) values (bold-italics values indicate the minimum AIC*c* value) for 10-fold dilutions of retropharyngeal lymph node tissue from white-tailed deer (WTD) #1, 4, and 11.

| **Model Selection and AICc Results** | | | | |
| --- | --- | --- | --- | --- |
| Prediction Model | Number of Parameters | Sample #1 AICc | Sample #4 AICc | Sample #11 AICc |
| $\boldsymbol{a\times}\boldsymbol{e}^{\boldsymbol{(-d \times ng)}}$ | 2 | -171.24 | -162.65 | -151.17 |
| $\boldsymbol{a+b \times}\boldsymbol{e}^{\boldsymbol{(-d \times ng)}}$ | 3 | -230.79 | -238.14 | -224.38 |
| $\boldsymbol{b\times}\boldsymbol{e}^{\boldsymbol{(-d \times ng)}}\boldsymbol{+c \times}\boldsymbol{e}^{\boldsymbol{(-f \times ng)}}$ | 4 | -239.16 | ***-238.52*** | ***-228.59*** |
| $\boldsymbol{a+b \times}\boldsymbol{e}^{\boldsymbol{(-d \times ng)}}\boldsymbol{+c \times}\boldsymbol{e}^{\boldsymbol{(-f \times ng)}}$ | 5 | ***-247.37*** | -235.83 | -225.82 |

**Supplementary Table S5.** Estimated parameters for the prediction models with the lowest Akaike information criterion corrected (AIC*c*) value for 10-fold dilutions of retropharyngeal lymph node tissue from WTD #1, 4, and 11. Parameter names in bold indicate names of variables in the prediction models.

| **Estimated Parameters** | | | | | |
| --- | --- | --- | --- | --- | --- |
| Sample # | Asymptote - **a** | Scale 1 - **b** | Scale 2 - **c** | Decay Rate 1 - **d** | Decay Rate 2 – **f** |
| 1 | 0.15050 | -0.04842 | 9.8922 e-6 | -0.09284 | 0.01059 |
| 4 | NA | 0.10758 | -0.10321 | 1.485 e-7 | 0.00012 |
| 11 | NA | 0.12772 | -0.11567 | -2.093 e-7 | 0.00013 |

**
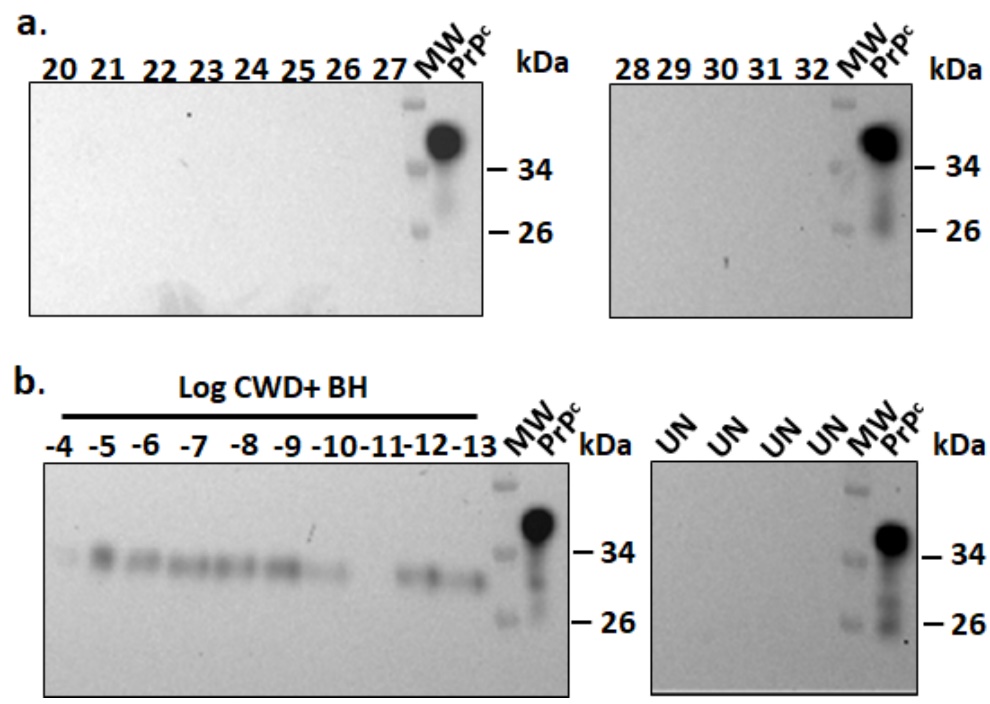
**

**Supplementary Figure S1. Additional testing of pooled tick samples collected from chronic wasting disease negative white-tailed deer (WTD) by the protein misfolding cyclic amplification (PMCA) assay. (a)** Tick homogenates prepared from 13 separate pooled tick samples collected from 13 CWD-negative WTD (ID 20-32); **(b)** serial dilutions of a CWD-positive (CWD+) brain homogenate (BH) (PMCA positive control), and unseeded (UN) or cellular prion (PrP^C^) (PMCA NCs). Samples analyzed in this figure were tested singularly and represent a third PMCA round. Numbers at the right of each panel represent molecular marker in kilodaltons (kDa).

**
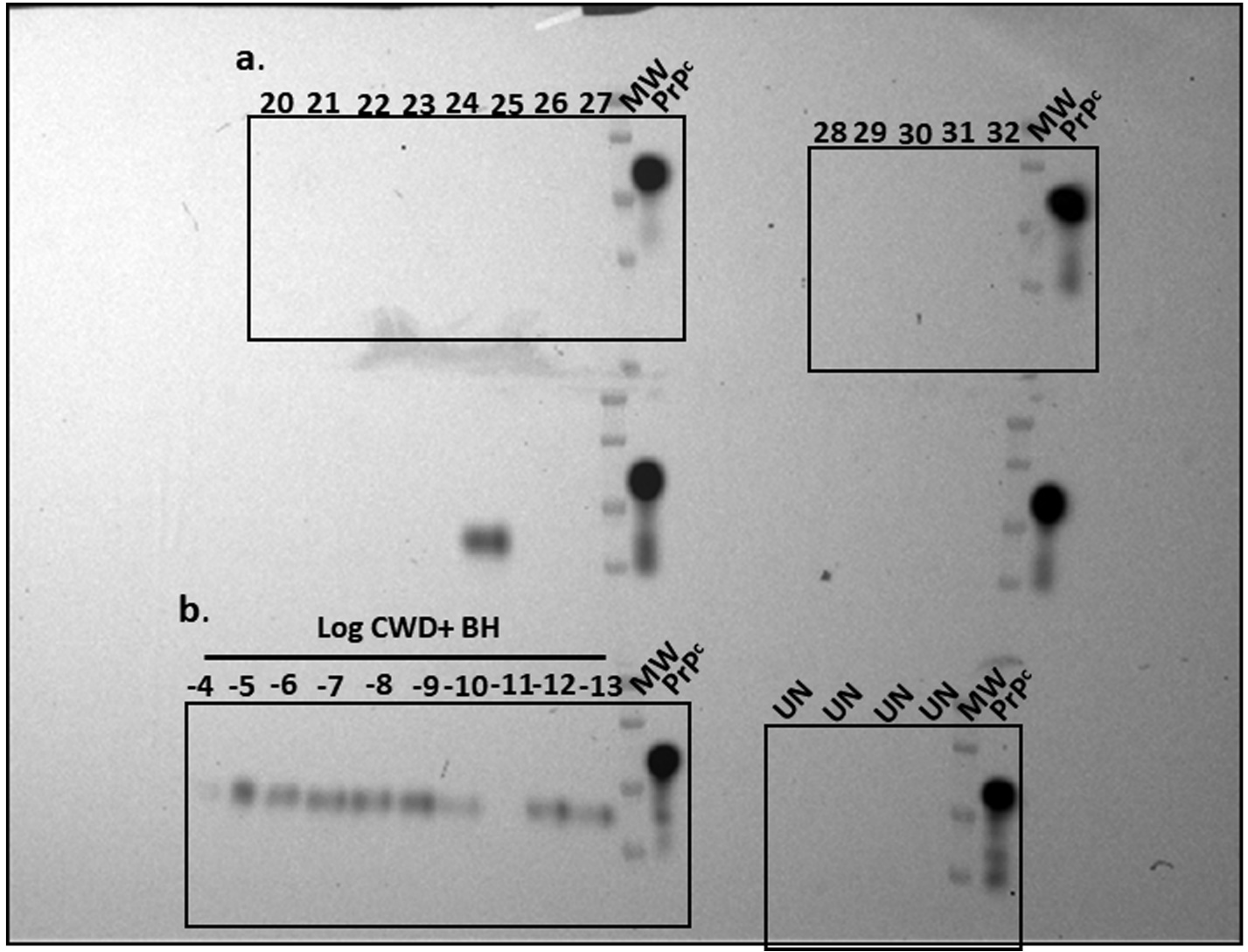
Supplementary Figure S2.** Uncropped Western blot images of pooled tick samples collected from chronic wasting disease (CWD)-negative white-tailed deer (WTD) by the protein misfolding cyclic amplification (PMCA) assay as depicted in Supplementary figure S1. **(a)** Tick homogenates prepared from 13 separate pooled tick samples collected from 13 CWD-negative WTD (ID 20-32); **(b)** serial dilutions of a CWD-positive (CWD+) brain homogenate (BH) (PMCA positive control), and unseeded (UN) or cellular prion (PrP^C^) (PMCA NCs). Samples analyzed in this figure were tested singularly and represent a third PMCA round. Uncropped blots pertinent to this study are boxed in with labels.

**
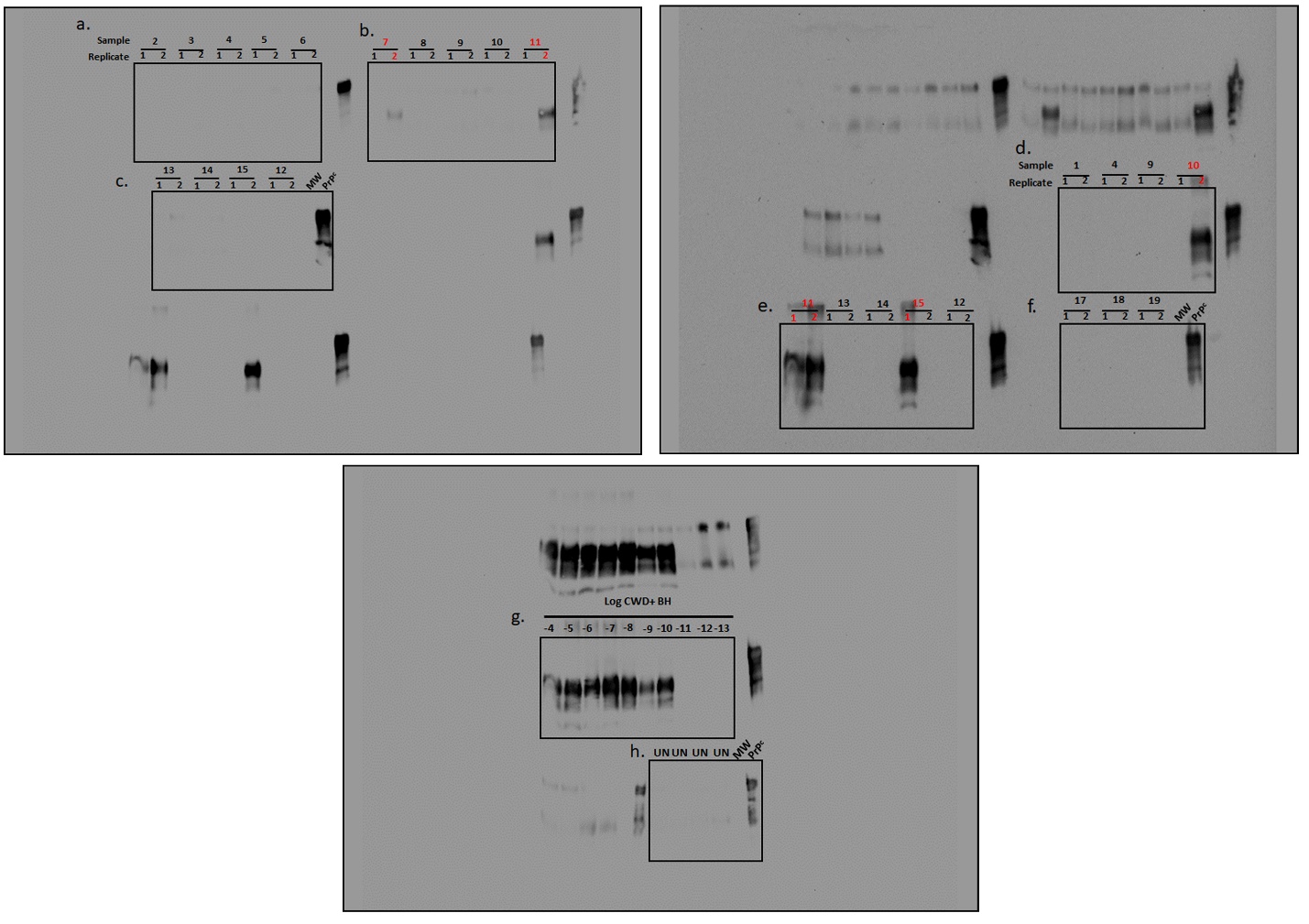
**

**Supplementary Figure S3.** Uncropped Western blot images of pooled tick samples collected from white-tailed deer (WTD) by the protein misfolding cyclic amplification (PMCA) assay as depicted in **(a-c)** “figure 2g”, **(d-f)** “figure 2h”, and **(g-h)** “figure 2i”. Uncropped blots pertinent to this study are boxed in with labels.
